# Potential of Wood-Rotting Fungi to Attack Polystyrene Sulfonate and Its Depolymerisation by *Gloeophyllum trabeum* via Hydroquinone-Driven Fenton Chemistry

**DOI:** 10.1371/journal.pone.0131773

**Published:** 2015-07-06

**Authors:** Martin C. Krueger, Ulrike Hofmann, Monika Moeder, Dietmar Schlosser

**Affiliations:** 1 Department Environmental Microbiology, Helmholtz Centre for Environmental Research—UFZ, Leipzig, Germany; 2 Department Analytical Chemistry, Helmholtz Centre for Environmental Research—UFZ, Leipzig, Germany; Genetics and Microbiology Research Group, SPAIN

## Abstract

Synthetic polymers often pose environmental hazards due to low biodegradation rates and resulting accumulation. In this study, a selection of wood-rotting fungi representing different lignocellulose decay types was screened for oxidative biodegradation of the polymer polystyrene sulfonate (PSS). Brown-rot basidiomycetes showed PSS depolymerisation of up to 50 % reduction in number-average molecular mass (M_n_) within 20 days. In-depth investigations with the most efficient depolymeriser, a *Gloeophyllum trabeum* strain, pointed at extracellular hydroquinone-driven Fenton chemistry responsible for depolymerisation. Detection of hydroxyl radicals present in the culture supernatants showed good compliance with depolymerisation over the time course of PSS degradation. 2,5-Dimethoxy-1,4-hydroquinone (2,5-DMHQ), which was detected in supernatants of active cultures via liquid chromatography and mass spectrometry, was demonstrated to drive the Fenton processes in *G*. *trabeum* cultures. Up to 80% reduction in M_n_ of PSS where observed when fungal cultures were additionally supplemented with 2,5-dimethoxy benzoquinone, the oxidized from of 2,5-DMHQ. Furthermore, 2,5-DMHQ could initiate the Fenton's reagent-mediated PSS depolymerisation in cell-free systems. In contrast, white-rot fungi were unable to cause substantial depolymerising effects despite the expression of lignin-modifying exo-enzymes. Detailed investigations with laccase from *Trametes versicolor* revealed that only in presence of certain redox mediators limited PSS depolymerisation occurred. Our results indicate that brown-rot fungi might be suitable organisms for the biodegradation of recalcitrant synthetic polymeric pollutants.

## Introduction

The release of synthetic polymers into the environment is of great concern, as many of them accumulate due to very slow degradation rates. Microplastics, particularly prominent in the marine environment, represent a striking example for polymers polluting the environment [1]. Depending on the nature of the polymer to be degraded, different physico-chemical and biological processes may contribute to the degradation process [2,3]. In many cases, oxidation processes play a key role since a large number of synthetic polymers feature unreactive carbon-carbon backbones. These are inert to hydrolysis and prevent rapid degradation.

Certain wood and litter decay fungi are promising candidates for the degradation of such polymers in the environment, since they have evolved strong oxidative, unspecific means to degrade the recalcitrant natural macromolecule lignin.

The degradative capacities of white-rot basidiomycetes have been thoroughly investigated. They are capable of a complete decomposition of lignin and its mineralisation into CO_2_ and H_2_O, which is initiated by an array of extracellular lignin-modifying enzymes. These include laccase, various peroxidases such as manganese peroxidase (MnP), versatile peroxidase (VP), lignin peroxidase (LiP), and perhaps also others [4–8]. The unspecific nature of this lignin-decomposing machinery also enables white-rot basidiomycetes to degrade a large range of organic environmental pollutants with low molecular masses [9] as well as various polymeric xenobiotics like phenol-formaldehyde resins [10,11], polyvinyl alcohol [12,13], and nylon [14–16]. Small organic compounds called redox mediators are known to greatly expand the substrate spectrum of laccases [17], which have been reported to decompose the synthetic polymer polyethylene in combination with such compounds [18].

Brown-rot basidiomycetes are not as well understood. Current genome studies suggest that they share a common white-rot ancestor with recent white-rot basidiomycetes, but have lost the ability to completely degrade lignin likely due to an evolutionary contraction of lignin-degrading peroxidases in the lineages leading to recent brown-rot species [19]. Nevertheless, brown-rot basidiomycetes are able to modify lignin to some extent [20,21]. Also, they have been demonstrated to degrade a variety of organic pollutants, including monomeric molecules like fluoroquinolone antibiotics [22] and chloro- and fluorophenols [23,24] as well as polymers like polyethylene oxide and polyvinyl alcohol [25,26]. Unspecific Fenton reactions driven by the redox cycling of Fe^III^-reducing hydroquinones and catechols like 2,5-dimethoxy-1,4-hydroquinone (2,5-DMHQ) and 4,5-dimethoxycatechol, which are produced as fungal metabolites, are believed to be the key mechanism employed by brown rot fungi for the degradation of wood as well as synthetic compounds [24,25,27–29]. A potential role of laccase has also been implied in these processes [30].

In this study, we investigated the ability of representative brown- and white-rot basidiomycetes to depolymerise the synthetic polymer polystyrene sulfonate (PSS). Furthermore, we have attempted to substantiate the mechanisms employed by basidiomycetes to decompose the compound. Due to the presence of sulfonic acid groups, PSS is water-soluble in contrast to many other polymers and therefore allowed us to circumvent bioavailability limitations created by solid, insoluble polymers such as the structurally similar polystyrene. PSS has widespread medical applications as an ion-exchange agent for the treatment of hyperkalaemia [31] as well as technical applications, e.g. in the creation of antistatic surfaces in combination with polythiophenes and for water treatment [32,33]. However, we are not aware of any report on its biodegradability.

Hence, we used PSS as a model compound for relatively unreactive synthetic polymers in this study. It proved highly resistant to degradation by white-rot fungi, but susceptible to the hydroquinone-driven Fenton chemistry employed by brown-rot fungi.

## Materials and Methods

### Chemicals

Poly(sodium 4-styrenesulfonate) (PSS; M_w_ ~70,000 Da) was obtained from Sigma-Aldrich (Munich, Germany). For some experiments with *G*. *trabeum* DSM 1398 indicated in the text, the low molecular mass fractions contained in the compound were eliminated by ultrafiltration using a 10 kDa cutoff membrane (Pall, Dreieich, Germany). All other chemicals were of analytical grade from various commercial sources (Sigma-Aldrich, Munich, Germany; ABCR, Karlsruhe, Germany; Merck, Schwalbach, Germany; Carl Roth, Karlsruhe, Germany; Th. Geyer, Renningen, Germany). 2,5-dimethoxy benzoquinone (2,5-DMBQ; ABCR, Karlsruhe, Germany) was reduced to 2,5-dimethoxy hydroquinone (2,5-DMHQ) as described by Kerem and colleagues [27]. 10 mM 2,5-DMBQ was dissolved in chloroform and thoroughly mixed with 500 mM sodium dithionite in water (half the chloroform volume). After phase separation, the chloroform phase was mostly evaporated under N_2_ stream. From the remaining chloroform phase (about 10% of the initial volume), 2,5-DMHQ was crystallised on ice. The second recrystallisation step was omitted as UPLC measurements (see below) did not show any significant impurities.

### Sources and maintenance of fungal strains

*Gloeophyllum trabeum* DSM 1398 and DSM 3087, *Gloeophyllum striatum* DSM 9592 and DSM 10335, *Stropharia rugosoannulata* DSM 11372, and *Trametes versicolor* DSM 11269 were obtained from the German Collection of Microorganisms and Cell Cultures (DSMZ, Braunschweig, Germany). Strain RT-2012-B2 was isolated from fruit bodies on rotting oak (*Quercus robur*) wood in the western lowland forest at Rosental, Leipzig, Germany, in autumn 2012. It was tentatively identified as *Trametes hirsuta*.

*Gloeophyllum* strains were maintained at 28°C on agar plates composed of a defined mineral medium devoid of a carbon, nitrogen, and phosphate source previously described. The composition was: 0.8 mM MgSO_4_, 0.2 mM CaCl_2_, 12 mM H_3_BO_3_, 0.4 mM CoSO_4_, 0.2 mM CuSO_4_, 0.04 mM (NH_4_)_6_Mo_7_O_24_, 2 mM MnSO_4_, and 0.4 mM ZnSO_4_ (pH 6), supplemented before inoculation with 20 μM FeSO_4_ (freshly prepared) and 1 ml (per liter of medium) of a vitamin stock solution containing (per liter) 20 mg of 4-aminobenzoic acid, 6 mg of D-(1)-biotin, 50 mg of DL-a-lipoic acid, 50 mg of nicotinamide, 10 mg of D-(1)-pantothenic acid calcium salt, 100 mg of pyridoxine x HCl, 50 mg of (2)-riboflavin, 20 mg of folic acid, 10 mg of thiamine x HCl, and 50 mg of cyanocobalamin ([22]; further on referred to as Wetzstein medium). For the agar plates, 10 g/l milled wheat straw (about 6 mm particle size) and 1.5% agar were added. All other strains except *T*. *hirsuta*, which was also maintained like the *Gloeophyllum* strains, were maintained on 1% malt extract agar plates (1% malt extract, 1.5% agar; pH 5.7) at 28°C.

### PSS degradation experiments using whole fungal cells

Prior to establishing liquid cultures, all strains were cultured on 1% malt extract agar plates at 28°C for one week. Three agar plugs of 1 cm diameter derived from fungal colonies on agar plates were used to inoculate preculture flasks containing 30 ml 5 g/l Oxoid CM 57 malt broth (Oxoid, Vienna, Austria). Precultures were incubated without agitation at 28°C in the dark for seven days.

For degradation experiments, liquid precultures were filtered over a sterile sieve. The retained mycelium was washed twice with 20 ml liquid degradation medium and then transferred to new flasks containing 30 ml degradation medium with 2 g/l PSS. For *Gloeophyllum* strains, Wetzstein medium (see above) was used as the degradation medium, while for the other strains a defined medium containing 56 mM glucose and 1.2 mM diammonium tartrate as carbon and nitrogen sources was employed as previously described for MnP production in *S*. *rugosoannulata* [34]. Control cultures were inactivated by the application of 1 g/l sodium azide. In some experiments with *G*. *trabeum* DSM 1398, various components expected to influence Fenton chemistry were added or removed from the Wetzstein medium to elucidate the mechanism of degradation as described in the text. Incubation was carried out at 28°C. For degradation experiments, triplicate cultures were always employed. For sampling, 1 ml of the supernatant was taken from the respective culture and used for analyses without further processing.

### PSS degradation experiments by cell-free Fenton reaction systems

To ascertain the role of hydroquinones in PSS depolymerisation, a biomimetic approach was developed. PSS (final concentration of 1 g/l) was added to 5 ml Wetzstein medium containing 20 μM FeSO_4_ and 500 μM 2,5-DMHQ, which was two more times applied at 500 μM during a total incubation period of 3 days (i.e. added after 1 and 2 days of incubation, respectively). In additional experiments, a single dose of 0.01% H_2_O_2_ was employed instead of 2,5-DMHQ at the beginning of the incubation. For controls, one of the components (2,5-DMHQ/H_2_O_2_ or FeSO_4_) was omitted. The reaction vials (always in triplicate) were incubated at 120 rpm and 28°C in the dark.

### Attempted PSS degradation by laccase-redox mediator systems

PSS (final concentration of 1 g/l) was added to 5 ml of 50 mM malonate buffer (pH 4.5) containing 10 U commercial laccase from *Trametes versicolor* (Sigma-Aldrich, Munich, Germany) and 0.25 mM of redox mediator, where the natural mediators acetosyringone, syringaldehyde and *p*-coumaric acid, and the synthetic mediators 2,2'-azino-bis(3-ethylbenzothiazoline-6-sulphonic acid) (ABTS), 1-hydroxybenzotriazole (1-HBT), (2,2,6,6-tetramethylpiperidin-1-yl)oxidanyl (TEMPO) and violouric acid were tested. The reaction was conducted at 120 rpm and 28°C on a rotary shaker in the dark (Infors HT, Infors, Bottmingen, Switzerland) over an incubation period of 4 days. Beyond their initial dosing as indicated above, redox mediators were further daily added at 0.25 mM to reaction mixtures. Negative controls contained the respective redox mediator and PSS, but no laccase. All experiments were conducted in triplicates.

### Analytical procedures

Size exclusion chromatography (SEC) was carried out using a Merck-Hitachi HPLC device previously described [35], which was equipped with a HEMA MCX 1000Å column (PSS Polymer Standards Service GmbH, Mainz, Germany) run at 40°C. The running buffer consisted of 5 g/l NaNO_3_, 2 g/l K_2_HPO_4_ and 20% acetonitrile in distilled water with the pH adjusted to 10. The flow rate was set to 1 ml/min. Samples were directly injected without prior treatment (injection volume always 100 μl). Chromatograms were recorded over a wavelength range from 220 to 600 nm (monitoring wavelength 250 nm). Molecular mass calibration for each run was achieved using the retention times of polystyrene sulfonate sodium salt molecular mass standards ranging from 0.208 to 79.9 kDa M_w_ (PSS Polymer Standards Service GmbH, Mainz, Germany) and the ExpDecay2 nonlinear fit function of the software OriginPro 9 (OriginLab, Northampton, USA). Number-average molecular masses (M_n_) and weight-average molecular masses (M_w_) were calculated from data of chromatograms recorded at 250 nm, using slices corresponding to elution time intervals of 1.6 seconds for single fractions. Only the polymeric fractions of PSS (>1.85 kDa) were considered in order to avoid potential interferences with unrelated low molecular mass compounds, e.g. fungal metabolites, redox mediators, and impurities in the original PSS.

The generation of thiobarbituric acid reactive substances (TBARS) was used to estimate the amount of extracellular hydroxyl radicals [36–38]. 2-Deoxyribose was added to 0.5 ml culture supernatant at a concentration of 2.8 mM and left to react in the dark for 120 min. Directly afterwards, 0.25 ml 2.8% (w/v) trichloroacetic acid and 0.25 ml 1% (w/v) thiobarbituric acid in 50 mM NaOH were added to the supernatant and incubated at 95°C for 15 min. After cooling, the absorbance at 530 nm was measured with a TECAN Genios plus plate reader (TECAN, Männedorf, Switzerland). Blanks, which contained water instead of deoxyribose, were subtracted to yield the final absorption values.

Quinones in culture supernatants were analysed by Ultra Performance Liquid Chromatography (UPLC). Aliquots from samples (3.3 μL) were directly subjected to an Acquity UPLC system (Waters, Eschborn, Germany) comprising of a PDA eλ photo diode array detector, a Binary Solvent Manager, and a Sample Manager which was equipped with an Acquity UPLC BEH C18 column (1.7 μm particle size; 2.1 × 50 mm; Waters) operated at 40°C. As mobile phases, two solvents were used: solvent A—10% methanol, pH 3.0 (gradient grade, pH adjusted with phosphoric acid) in deionised water (Q-Gard 2, Millipore, Schwalbach, Germany); solvent B—100% methanol, pH 3.0. The following elution profile was applied: isocratic elution at 20% B for 0.14 min; linear increase to 100% B until 2.8 min; isocratic elution at 100% B until 3.2 min; linear decrease to 20% B until 3.25 min; isocratic elution at 20% B until 3.5 min. Flow rate was adjusted to 0.5 ml/min. A wavelength range from 220 to 400 nm was recorded (detection wavelength: 278 nm) [39]. External standards of commercial 2,5-DMBQ and self-produced 2,5-DMHQ (see above) were used for calibration.

For verification, gas chromatography-mass spectrometry (GC-MS) was applied after extraction of 1.5 ml culture supernatant from day 10 with 3 x 200 μL chloroform (GC-grade, Sigma Aldrich, Seelze, Germany). The extracts were unified and evaporated at a gentle stream of nitrogen gas to a final volume of 1 ml. For analysis, 1 μL were injected splitless (1 min vent time). The analysis was performed with a GC-MSD system (Agilent Technologies, Waldbronn, Germany). Experimental details on GC-MS analysis are described in the S1 File.

Lignin-modifying enzyme activities were analysed spectrophotometrically [40]. Oxidation of 2 mM 2,2'-azino-bis(3-ethylbenzothiazoline-6-sulfonic acid) (ABTS); ε_436_ = 29.3 mM^-1^ cm^-1^) in 50 mM sodium malonate buffer at pH 4.5 was determined with a TECAN Genios plus plate reader (TECAN, Männedorf, Switzerland). Laccase (EC 1.10.3.2) and manganese-independent peroxidase (MiP; peroxidase, EC 1.11.1.7) activities were measured in the presence of 1 mM EDTA intended to prevent interferences caused by traces of manganese possibly present in samples. For peroxidases, 100 μM H_2_O_2_ was additionally included. For manganese peroxidase (MnP; EC 1.11.1.13), EDTA was replaced by 500 μM MnCl_2_. MiP was corrected for laccase activities, and MnP was corrected for MiP and laccase activities. One enzyme unit (U) corresponds to 1 μmol ABTS oxidised per minute.

## Results

### Screening of wood-rotting fungi for PSS depolymerisation

**Fig 1:**
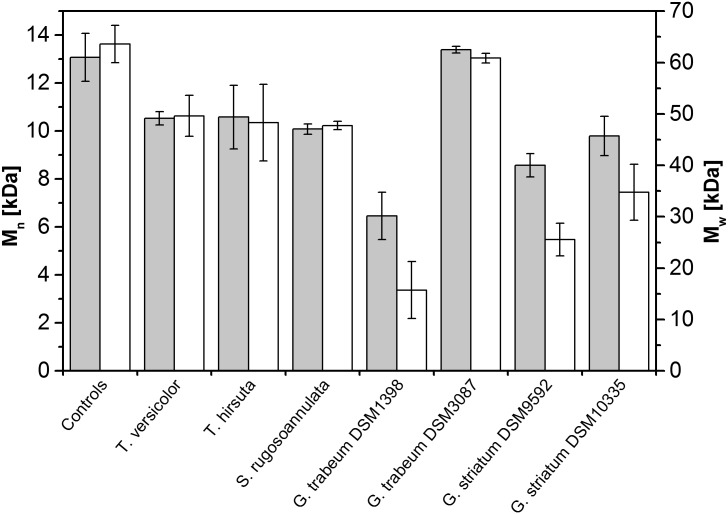
Molecular masses of PSS after fungal treatment for 20 days. Grey: M_n_; white: M_w_. Corresponding controls were inactivated with sodium azide. Bars represent means ± standard deviation for triplicate cultures.

Liquid cultures run for 20 days were used to assess the general potential of the fungal strains to depolymerise PSS using SEC analysis. The M_n_ and M_w_ of PSS at the time of harvest are depicted in Fig 1.

The results for the brown-rot strains were non-uniform. *G*. *striatum* DSM 9592 and *G*. *trabeum* DSM 1398 caused substantial depolymerisation, leading to final M_n_ values of 8.3 ± 0.5 and 6.5 ± 1 kDa, respectively. *G*. *striatum* DSM 10335 reduced M_n_ value only moderately to 10 ± 0.7 kDa compared to the inactivated controls (12.9 ± 1 kDa), whereas *G*. *trabeum* DSM 3087 did not cause any measurable depolymerisation. The active *Gloeophyllum* strains also caused substantial reductions of the M_w_ values, i.e. from 63.5 ± 3.7 kDa to 34.9 ± 5.4, 25.4 ± 3.2, and 15.7 ± 5.5 kDa in case of *G*. *striatum* DSM 10335, *G*. *striatum* DSM 9592, and *G*. *trabeum* DSM 1398, respectively. No activities of any lignin-modifying enzyme could be detected in the supernatants of the cultures at the time of harvest and, in case of *G*. *trabeum* DSM 1398, where samples were repeatedly taken during the cultivation, also during the entire cultivation time.

All three white-rot fungal strains displayed only weakly reduced M_n_ values of 10.1 ± 0.3 kDa for *T*. *versicolor*, 9.3 ± 0.2 kDa for *S*. *rugosoannulata* and 10 ± 1.3 kDa for *T*. *hirsuta*, compared with 12.9 ± 1 kDa recorded in the inactivated controls (Fig 1). M_w_ was reduced only weakly to about 47–49 kDa. Lignin-modifying enzyme activities of up to 114 U/l manganese peroxidase and 28 U/l Mn-independent peroxidase in case of *T*. *hirsuta* were detected in the culture supernatants after 20 days. No lignin-modifying enzyme activities were observed for *T*. *versicolor* at the end of cultivation in glucose-containing degradation medium. However, when placed in Wetzstein medium, it produced up to 600 U/l laccase, but without any effect on PSS depolymerisation (data not shown). *S*. *rugosoannulata* never displayed any lignin-modifying enzyme activities in the presence of PSS, but expressed substantial quantities of up to 117 U/l manganese peroxidase when PSS was absent from the culture medium.

Because of its most efficient performance concerning PSS depolymerisation, additional experiments concentrated on *G*. *trabeum* DSM 1398.

### Mechanism of PSS depolymerisation by *G*. *trabeum* DSM 1398

**Fig 2:**
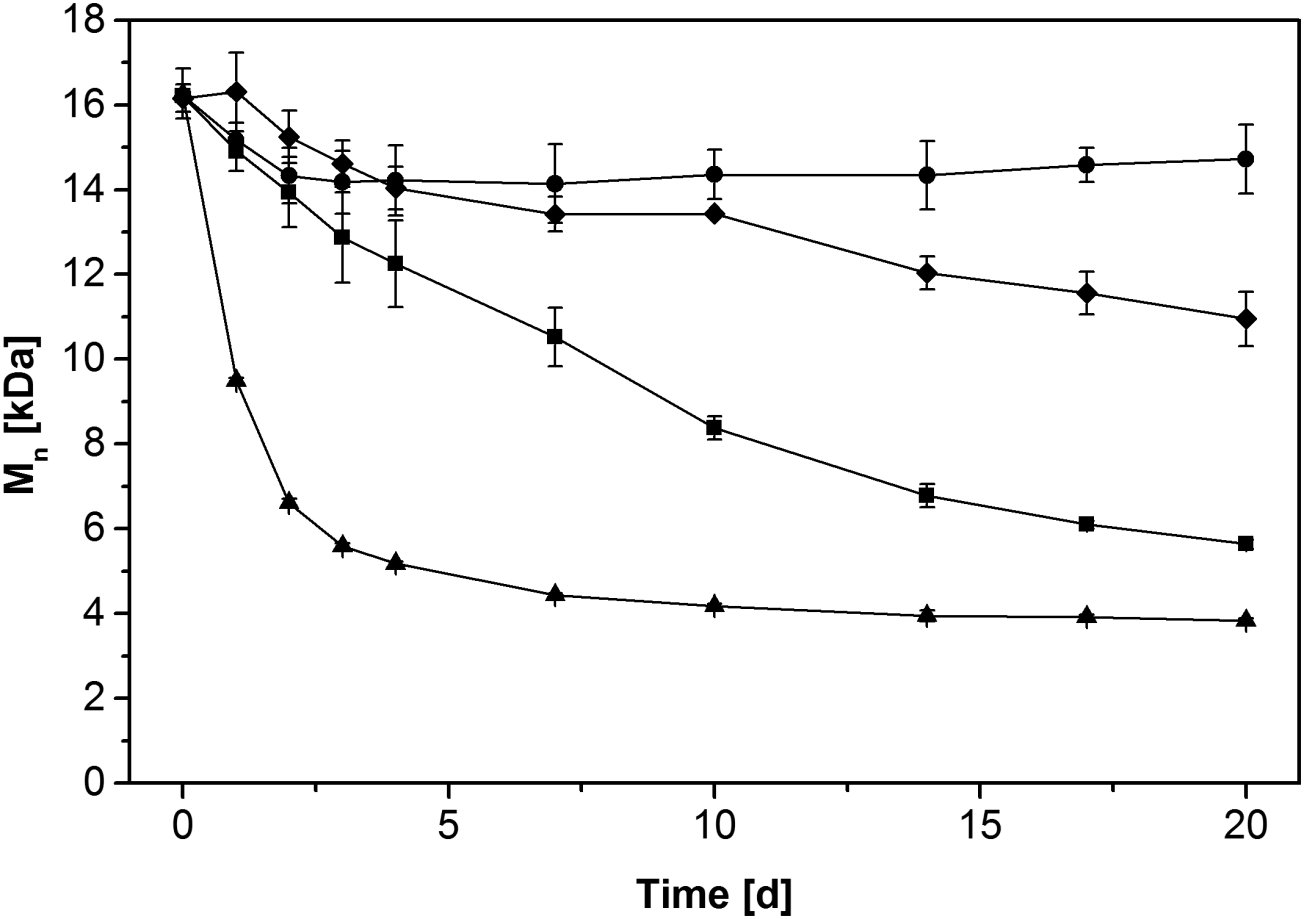
Time course of PSS depolymerisation by whole cells *G*. *trabeum* DSM 1398. Cultures in regular Wetzstein medium (■) were compared to those supplemented with 500 μM 2,5-DMBQ (▲), without iron (♦), and cultures inactivated by sodium azide (control; ●). Symbols represent means ± standard deviations for triplicate cultures; standard deviations smaller than symbol size are not shown.

Past research on Fenton chemistry-based degradative mechanisms of brown-rot fungi has yielded insight into various factors such as the presence of additional hydroquinones or mannitol, which either enhance or reduce their activity [24,27]. Several related conditions were tested with *G*. *trabeum* DSM 1398 in order to substantiate whether Fenton chemistry is operative during PSS depolymerisation by brown-rot fungi. Table 1 depicts the effects of these conditions on the molecular mass of PSS remaining after 20 days in liquid culture relative to the controls. The presence of the iron chelator oxalate, the known hydroxyl radical scavenger mannitol, as well as the omission of 20 μM FeSO_4_ from Wetzstein medium severely inhibited PSS depolymerisation. In contrast to these observations, the addition of 500 μM 2,5-dimethoxy-1,4-benzoquinone (2,5-DMBQ), naturally employed by *Gloeophyllum* to drive its Fenton chemistry [41], significantly speeded up PSS depolymerisation as depicted in Fig 2. To a lesser extent, it also reduced the final molecular mass of PSS fragments compared to non-supplemented cultures.

**Table 1:** Effect of varied conditions on PSS depolymerisation by *G*. *trabeum* .

Reaction conditions	Relative M_n_ of PSS (% of control)^1^
Regular Wetzstein medium	47 ± 3.8
+500 μM 2,5-DMBQ	36.8 ± 3.8
FeSO4 omitted	79.9 ± 16.2
+5 mM Na oxalate	78.4 ± 4.6
+56 mM mannitol	87.1 ± 5.7

Samples taken from PSS-containing *G*. *trabeum* DSM 1398 after 20 days of cultivation were compared for the relative M_n_ value of remaining PSS polymer.

^1^ The M_n_ value in NaN_3_-inactivated cultures serving as controls (14.2 ± 0.5 kDa) was set to 100%. Data represent means ± standard deviations for triplicate cultures.

**Fig 3:**
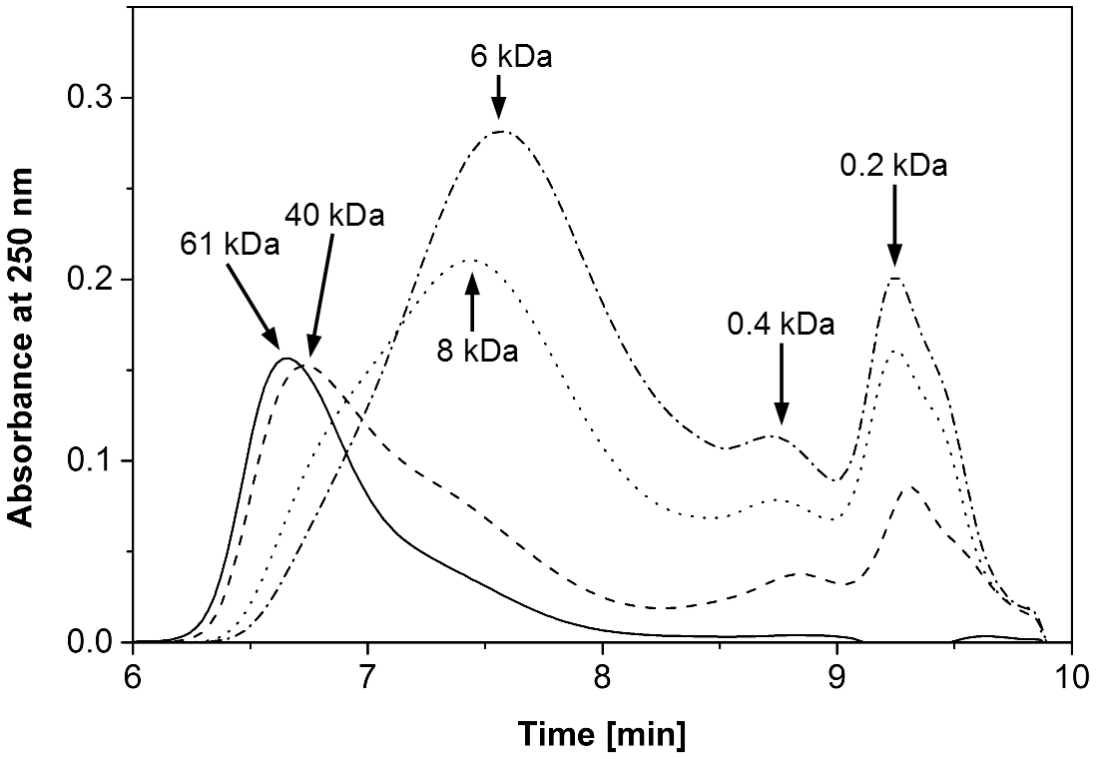
SEC elution profiles of samples from culture supernatants. Samples were taken over the course of incubation of a single representative *G*. *trabeum* culture containing ultrafiltrated PSS in regular Wetzstein medium. Samples from the start of cultivation ( — ), and from culture day 7 ( - - ), 14 (∙ ∙ ∙), and 20 ( - ∙ - ) were analysed.

Further insight into the depolymerisation mechanism was gained from comparisons of SEC chromatograms recorded at different times of cultivation (Fig 3). With increasing time, the peak corresponding to the major PSS fraction continuously moved to longer retention times indicating lower molecular masses. Concomitantly, two new peaks with intensities increasing over time appeared at retention times in the range of mono- and dimeric compounds (i.e. approximately 200 and 400 Da). The increase in intensities of these peaks apparently was a continuous process (S1 Fig). Unfortunately, certain fractions of the partially degraded PSS showed a considerably higher absorbance than the undegraded polymer at around 250 nm, which hampered a direct comparison and quantification (compare especially S2A and S2B Fig).

**Fig 4:**
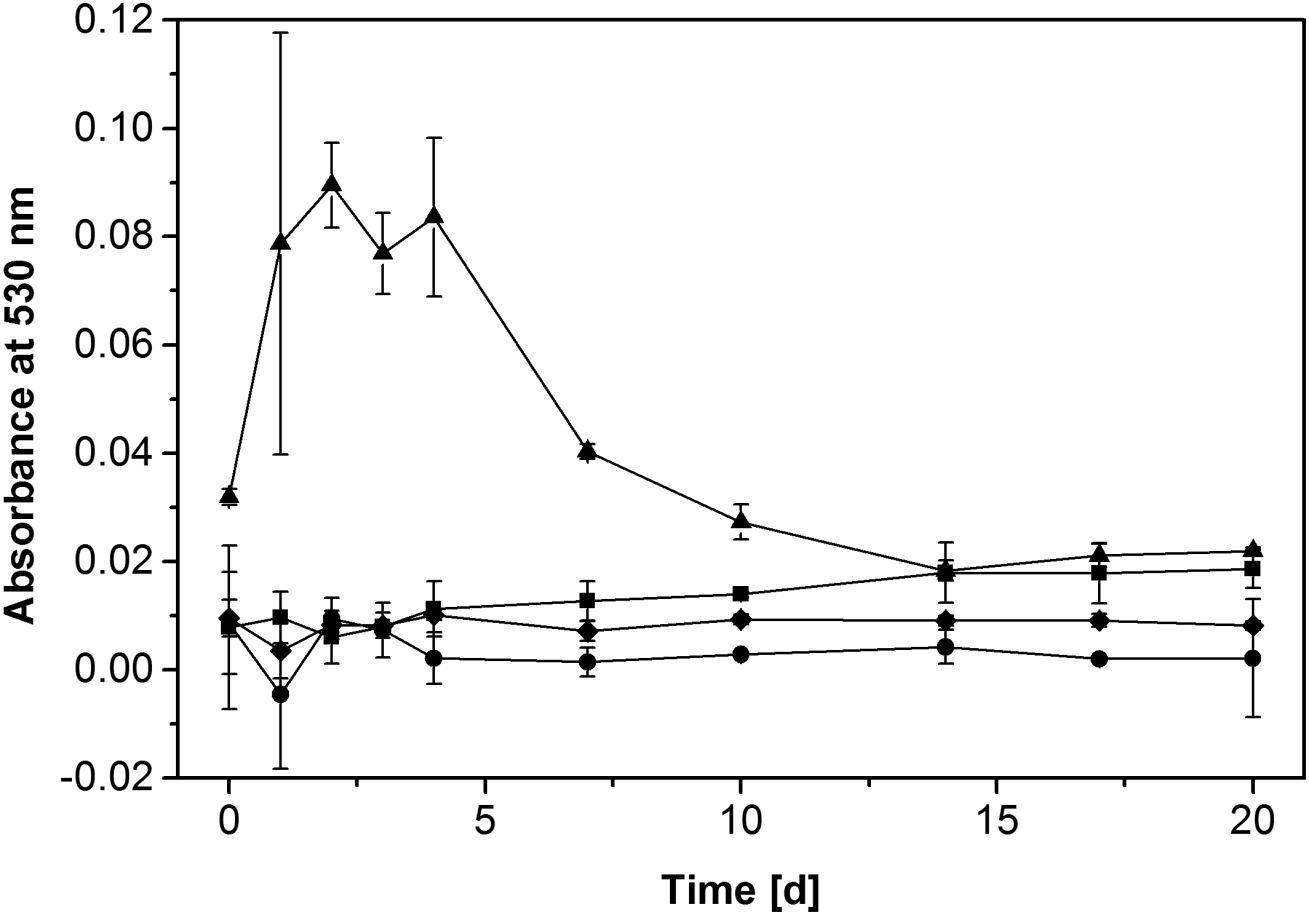
Hydroxyl radical generation by *G*. *trabeum* under varied culture conditions. Production of TBARS from 2-deoxyribose monitored at 530 nm was used as an indicator. Conditions shown: culture in regular Wetzstein medium (■); azide-inactivated (●); no iron (♦); quinone-supplemented (▲). Symbols represent means ± standard deviation for triplicate cultures; standard deviations smaller than symbol size are not shown.

Production of TBARS was used as a proxy for the production of hydroxyl radicals under four different culture conditions (Fig 4). Sodium azide-inactivated cultures showed practically no absorbance from deoxyribose-derived TBARS. Likewise, cultures deprived of iron showed an only weakly increased absorbance during the whole time course of cultivation. In contrast, quinone-supplemented cultures showed a clearly stimulated TBARS production during the first four days of cultivation and dropped to the level of regular cultures afterwards. Regular fungal cultures (i.e. those active cultures containing iron and PSS, but no other supplements) produced only low levels of TBARS during the first days of cultivation, but increased TBARS generation to an intermediate level after approximately one week and maintained that level until the end of the experiment at 20 days.

**Fig 5:**
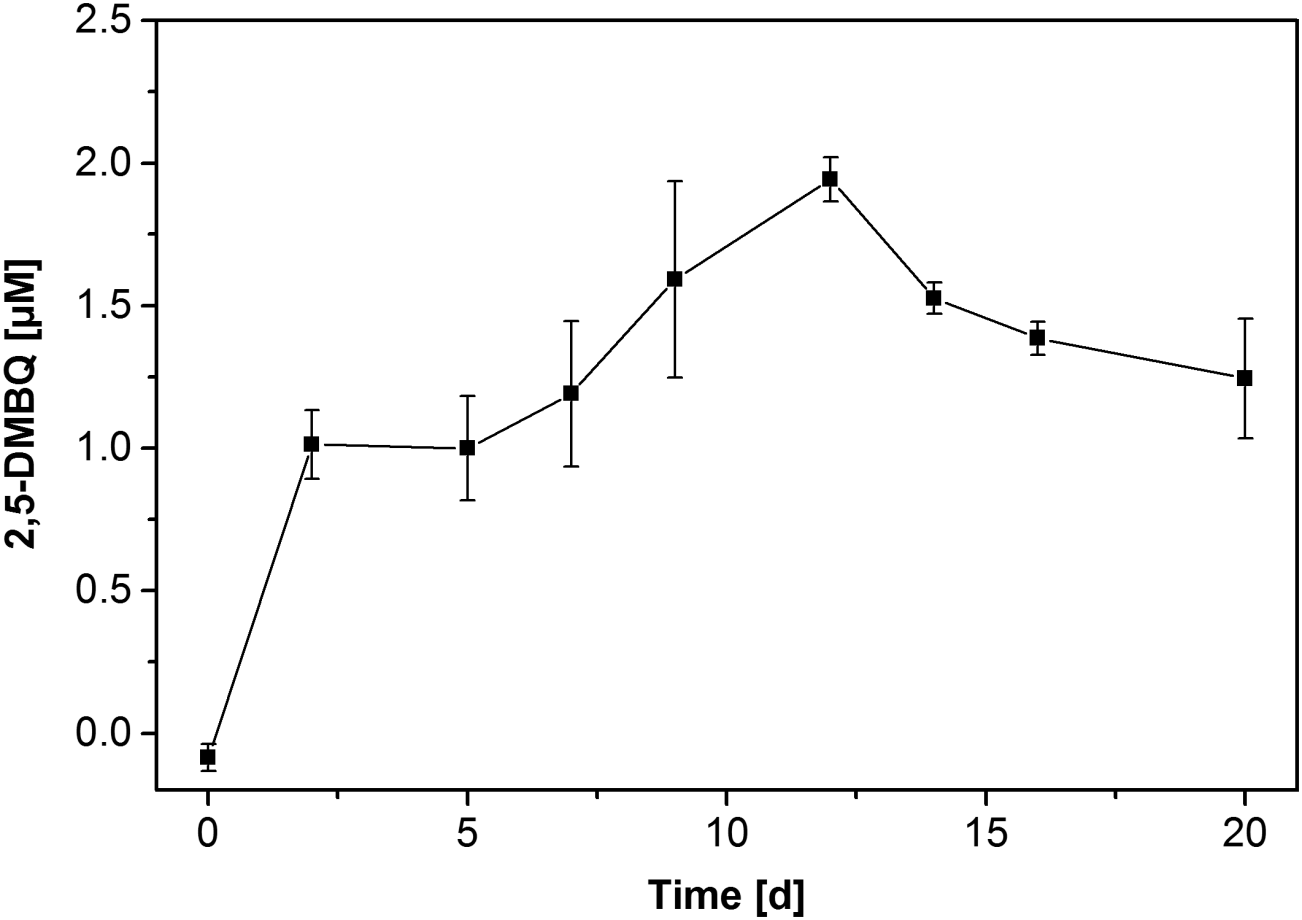
Concentration of 2,5-DMBQ in supernatants of actively depolymerising *G*. *trabeum* cultures in regular Wetzstein medium. Symbols represent means ± standard deviation of triplicate cultures.

**Fig 6:**
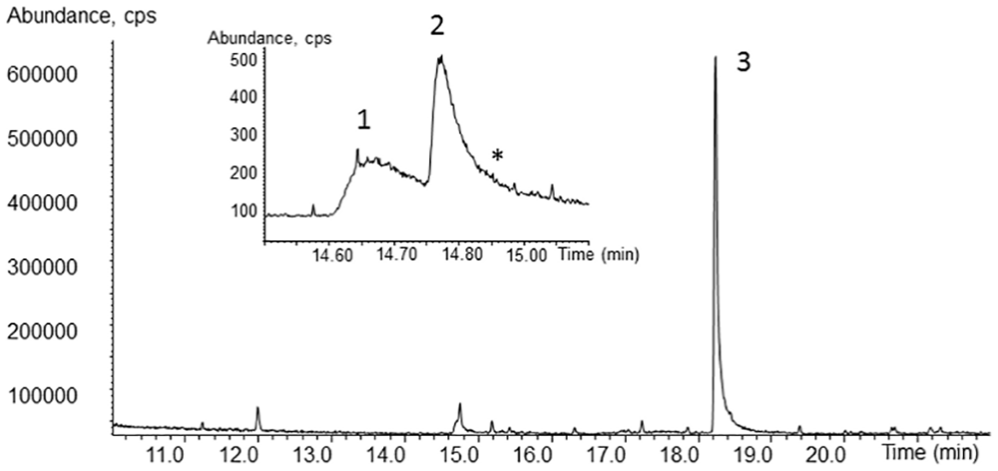
Total ion current chromatogram of the extract obtained from culture supernatant. The sample was taken from a regular culture after 10 days of incubation. The magnified sector indicates the 2,5-DMHQ (no. 1) and the 2,5-DMHQ monoacetate (no. 2) as well as the inferred 2,5-DMBQ (*). The signal no. 3 has the molecular mass 190 g/mol.

Fungal production of quinones potentially contributing to extracellular Fenton chemistry in *G*. *trabeum* [27,41] was evidenced by analysis of fungal culture supernatants, using UPLC coupled to UV/vis detection where 2,5-DMBQ shows a much stronger absorption in the UV range than 2,5-DMHQ does. 2,5-DMBQ present in fungal culture supernatants eluted around 0.44–0.45 min (authentic 2,5-DMBQ used as a standard: 0.445 min) and produced a distinct peak in the chromatograms. The UV/vis spectrum of this peak was in good agreement with authentic 2,5-DMBQ standards (maximum absorbance for both compounds at approximately 282 nm). In Fig 5, the development of 2,5-DMBQ concentration over the time course of a cultivation in regular Wetzstein medium is shown. 2,5-DMBQ concentration increased during the first half of cultivation and slightly declined thereafter. 2,5-DMHQ could not be detected in any sample via UPLC, most likely due to its relatively weak absorbance. However, after extraction of the aqueous samples and enrichment of the analytes, 2,5-DMHQ could be detected by GC-MS. The GC-MS analysis of an extract prepared from a sample after 10 days of incubation provided the chromatogram shown in Fig 6. The cut in Fig 6 exhibits the magnified region of the chromatogram indicating the 2,5-DMHQ (signal no.1) and the monoacetate of 2,5-DMHQ (no.2), which is formed during the fungal treatment. The structure of the monoacetylated 2,5-DMHQ was proven by a synthesised reference compound. The corresponding mass spectra and GC-MS data are presented in the S1 File. 2,5-DMBQ („*”in Fig 6) seems to co-elute with 2,5-DMHQ monoacetate but due to the inferred mass spectra, an unambiguous identification in full scan mode was difficult.

The compound with a molecular mass of 190 g/mol predominates the GC-MS chromatogram (Fig 6, signal no. 3). After comparing with mass spectra of the NIST02-library, the mass spectrum of 1-[5-(2-furanylmethyl)-2-furanyl]-ethanone exhibited the best match (77%). This substance has already been observed in *Gloeophyllum striatum* and seems to be produced by the genus *Gloeophyllum* [24]. The corresponding mass spectrum is given in the S1 File.

The involvement of the 2,5-DMHQ/2,5-DMBQ couple in Fenton reaction-driven PSS depolymerisation was established using cell-free reaction mixtures in fresh Wetzstein medium. PSS was depolymerised by Fenton reactions driven by 2,5-DMHQ as well as by H_2_O_2_. The molecular masses of PSS and its formed degradation products were analysed by SEC. Untreated ultrafiltrated PSS showed a M_n_ value of 19.5 ± 0.5 kDa. Treatment with 2,5-DMHQ and H_2_O_2_ reduced this value to 5.5 ± 0.1 and 3.8 ± 0.6 kDa, respectively. No effects were observed when DMHQ or H_2_O_2_ were omitted.

Furthermore, we used a biomimetic approach to investigate whether the ion exchange properties of PSS played a role in its degradation. An excess of either MgCl_2_ or CaCl_2_ at 20 mM caused inhibition of depolymerisation in a biomimetic system composed of a total of 1.5 mM of the commercially available 2,6-DMHQ (added at 500 μM at the start of incubation and the following two days, respectively) and Wetzstein medium containing 20 μM FeSO_4,_ and resulted in M_n_ values of PSS of 11 ± 0.2 and 10.1 ± 0.3 kDa for MgCl_2_ and CaCl_2_, respectively, compared with 8.3 ± 0.4 kDa observed in their absence.

### Degradation of PSS by laccase-mediator systems (LMS)

As the depolymerisation of synthetic polymers like nylon and polyethylene in presence of redox mediators has been described for laccases from white-rot fungi [18], the degradation of PSS by laccase with and without the use of a redox mediator was investigated. Table 2 shows the relative molecular masses of PSS after the treatment with laccase and different redox mediators. After 4 days of laccase treatment without mediator, the molecular mass of PSS did not decrease, suggesting that laccase alone has no effect on PSS. PSS degradation products with lowered M_n_ values were formed when the natural mediators *p*-coumaric acid, syringaldehyde, and the synthetic mediator 1-HBT were additionally included, respectively. Further mediators tested were not effective (Table 2).

**Table 2:** Relative molecular mass of PSS after three days of LMS treatment measured by SEC .^1^

Redox mediator	Relative M_n_ of PSS (% of initial)^2^
No redox mediator	99.8 ± 9.2
ABTS	97.0 ± 4.9
1-HBT	79.8 ± 1.5
TEMPO	101.8 ± 2.8
Violouric acid	100.1 ± 5.2
Acetosyringone	94.1 ± 3.1
Syringaldehyde	86.3 ± 3.4
*p*-coumaric acid	75.8 ± 1.1

^1^ The M_n_ values before start of the LMS treatment (ranging from 12.1±0.2 to 13.6±0.4 kDa) were set to 100%, respectively.

^2^ Data represent means ± standard deviations for triplicate cultures.

## Discussion

In this study, PSS was used as an easily bioavailable model to assess the degradative capacities of wood-decaying basidiomycetes in depolymerising unreactive synthetic polymers. We found that PSS was a highly recalcitrant compound, as white-rot fungi, which are effective degraders of a wide range of organopollutants [9], were unable to cause substantial depolymerising effects. By contrast, brown-rot basidiomycetes were capable of initiating depolymerisation. The remarkably different results with the four *Gloeophyllum* strains (Fig 1) might have been caused by different physiological adaptations to Wetzstein medium or by different temporal degradation activity patterns, as already observed by other authors [42].

Among the *Gloeophyllum* strains, *G*. *trabeum* DSM 1398 was selected for detailed investigation of the mechanisms underlying PSS depolymerisation as it had caused the strongest depolymerisation effects in the general screening for PSS depolymerisation (Fig 1). Previously, it has been suggested that brown-rot fungi of the genus *Gloeophyllum* drive extracellular Fenton chemistry through the redox cycling of hydroquinones, which is employed as a degradative machinery by these organisms [27,28,41,43]. Our results are in good accordance with this model.

PSS depolymerisation appeared to be a fairly unspecific process, as the polymer peak continuously moved towards lower molecular masses in the SEC measurements (Fig 3). This observation indicates that polymer chain scissions happened randomly across the entire chain, instead of just in specific positions, which would have caused a depolymerisation pattern characterised by the immediate appearance of distinct new peaks. Concomitant exo-scissions at the ends of polymer chains would explain the appearance of dimeric and monomeric molecule fragments, which likely accumulated due to a lacking or only slow further degradation. Although it is known that *Gloeophyllum* can completely mineralise xenobiotics and synthetic polymers like polyethylene glycol, the amount of mineralisation has been reported to be rather low when confronted with high concentrations of the polymer [25] comparable to the ones used in our experiments. Additionally, accumulation of fungal metabolites, possibly including DMHQ condensation products, may have contributed to the accumulation of low molecular mass fragments that could not be further resolved by the column. We therefore decided to focus SEC measurements on the polymeric fractions of PSS, in order to minimise analytical bias potentially caused by the aforementioned confounding factors.

With regard to components of the medium (see Table 1), freely available dissolved iron was necessary for depolymerisation activity and oxalate, which forms Fe^III^ complexes unavailable for reduction by hydroquinones [24], significantly inhibited the process. A high concentration of mannitol caused even more severe inhibition. All these observations point to hydroxyl radicals as the prime agents of PSS backbone scission, which are formed in Fenton reactions when Fe^II^ is oxidised by hydrogen peroxide. These radicals are the strongest oxidants known in biology, with a redox potential of +2.7 V [44], and can therefore easily oxidise most organic substrates. Hence, mannitol would compete with PSS for hydroxyl radicals and inhibit degradation as observed.

Among synthetic polymers, it is likely that PSS is an especially suitable target for degradation by such Fenton chemistry due to its ion exchange properties, as it has been suggested that sulfonate groups on previously sulfonated polystyrene might hold the reactive Fe^II^ ions in relatively close proximity to the polymer backbone [45]. Our observation of inhibited PSS depolymerisation in the presence of an excess of redox-inactive bivalent Mg^2+^ and Ca^2+^ ions supports this theory.

PSS has already been reported to be rather vulnerable to degradation by hydroxyl radicals [46,47]. Previously described degradation products resulting from pulse radiolysis-initiated hydroxyl radical attack on PSS [47] also offer an explanation for the observed increase in absorbance after degradation (S2 Fig). PSS oxidation by hydroxyl radicals may involve addition reactions resulting in aromatic ring substitution, and also tertiary benzyl radical formation via secondary decay reactions. Tertiary benzyl radicals may also be formed via hydrogen abstraction, and give rise to backbone scission and the formation of a variety of structures with additional conjugated double bonds [47].

Addition of 2,5-DMBQ to fungal cultures strongly enhanced the velocity of the depolymerisation process (Fig 2), most likely by providing a higher initial amount of quinone for redox cycling. These observations point to fungal production and/or excretion of hydroquinones as a limiting step for PSS depolymerisation under the regular liquid culture conditions applied in these experiments, while reduction of the benzoquinone to its corresponding hydroquinone by the fungal mycelium proceeds rapidly. As such, the results are in good agreement with earlier observations [27] as well as with our TBARS measurements, which showed a high peak of activity during the first days of cultivation and then rapidly decreasing activity in quinone-supplemented cultures (Fig 4). The slow depolymerisation rates after the initial four days point at a declining fungal capacity for quinone cycling, which does not come as a surprise given the low amount of biomass and the total lack of any carbon and energy source in Wetzstein medium leading to fungal storage compound depletion over time.

The hypothesis of the crucial role of 2,5-DMHQ is supported by the detection of its corresponding benzoquinone in *G*. *trabeum* culture supernatants as well as the abiotic PSS depolymerisation produced by a cell-free Fe^II^-DMHQ system, which produced a depolymerisation pattern very similar to that of *G*. *trabeum*. The rather low amounts of measurable quinone were likely to have a fast turnover, as 2,5-DMHQ oxidation proceeds rapidly in the presence of iron salts and molecular oxygen [41].

In contrast to the brown-rot fungi, white-rot basidiomycetes were not able to substantially depolymerise PSS, which may have been caused by several factors. In case of *S*. *rugosoannulata*, PSS appeared to completely inhibit production of laccase and MnP. Reasons for this might be the deprivation of the fungus of necessary inducing metal ions due to the ion exchange properties of PSS, or some other as yet unknown interaction by the fungus with either the polymer or low molecular weight fractions of the PSS solution. However, both *T*. *versicolor* and *T*. *hirsuta* did not cause substantial depolymerisation of PSS despite high activities of laccase and peroxidases, respectively. This may indicate that lignin-modifying enzymes *in vivo* are not able to depolymerise PSS, and perhaps also do not efficiently decompose other unreactive synthetic polymers. We suggest a combination of two compounding factors as the reason for this observation: first, enzymes acting through small molecular intermediates like manganese peroxidase probably lack the oxidative power to break the unreactive C-C and C-H bonds of the polymer backbone. Indeed, the only reports on the degradation of a synthetic linear C-C polymer by white-rot fungi we are aware of concern polyvinyl alcohol degradation by *Phanaerochaete chrysosporium* and *Pycnoporus cinnabarinus* [12,13,48], which already contains reactive hydroxyl groups directly on its backbone. Second, steric reasons might play a role for enzymes that would directly oxidise their substrate, preventing the large PSS polymer chain from entering any substrate binding site that is not located directly at the enzyme surface and widely open to its surroundings.

Our experiments with *T*. *versicolor* laccase further reinforced these findings. Previously, a combination of the redox mediator HBT and laccase has been reported to cause significant depolymerisation of the unreactive polymer polyethylene [18], so we assumed that it might also work for PSS. HBT is known to be a particularly effective laccase mediator [17] and indeed, it caused moderate depolymerisation of PSS, while most other natural and synthetic redox mediators employed (with the exception of *p*-coumaric acid and, to some extent, syringaldehyde) did not cause depolymerisation greater than 10% and were therefore deemed ineffective. These results demonstrate that PSS depolymerisation by the laccase-mediator system is, in principle, possible. However, our results may indicate only little relevance under environmental conditions due to the apparent inability of most mediators tested to depolymerise PSS and the relatively poor performance of the active mediators.

Although we did not test any further lignin-modifying enzymes *in vitro*, it appears that manganese peroxidase is incapable of initiating PSS depolymerisation and that unspecific peroxygenase from *Agrocybe aegerita* can cause slight depolymerising effects only when supported by Fenton chemistry (Kluge and Liers, personal communication).

In conclusion, we have demonstrated that the synthetic polymer PSS can be depolymerised through the action of brown-rot fungi. Fenton chemistry with a central role of hydroquinones produced by the fungi was found to be the most likely agent of biodegradation. In future studies, we will investigate whether this degradation mechanism can also be applied to even less reactive, solid polymers like polystyrene.

## Supporting Information

S1 Fig Relative decline in the original peak and emergence of mono- and dimeric peaks. (BMP)

S2 Fig UV/vis spectra of undegraded PSS and degradation products after 20 days. (BMP)

S1 File Supporting MS data containing detailed experimental MS procedures and additional MS data. (DOCX)
