# Supplementary figures and images for: Potential of Wood-Rotting Fungi to Attack Polystyrene Sulfonate and Its Depolymerisation by Gloeophyllum trabeum via Hydroquinone-Driven Fenton Chemistry
Source: PLoS One. 2015 Jul 6;10(7):e0131773. doi: 10.1371/journal.pone.0131773 (PMC4493105; doi:10.1371/journal.pone.0131773)

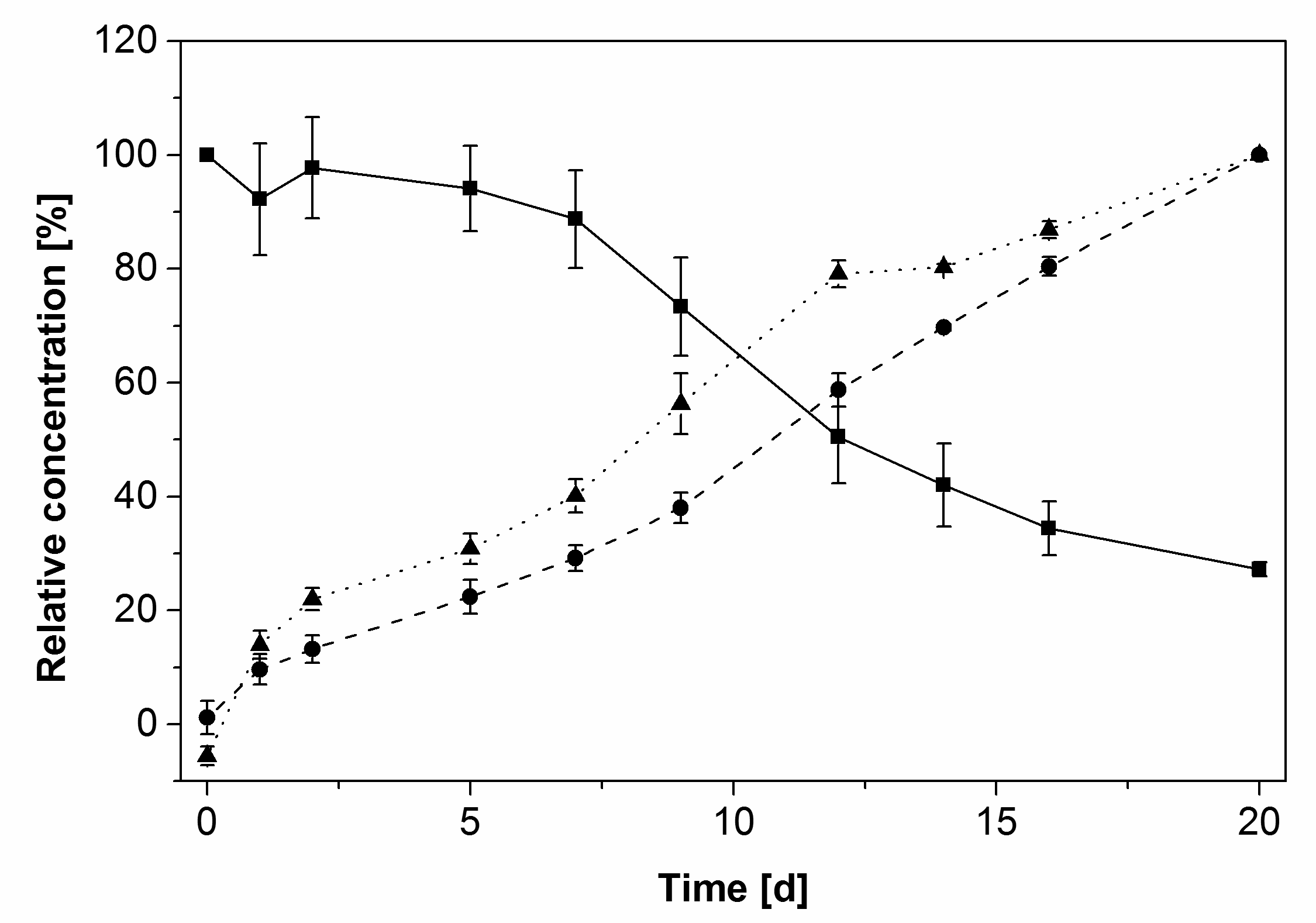

Supplement: S1 Fig — (BMP) [file pone.0131773.s001.bmp]

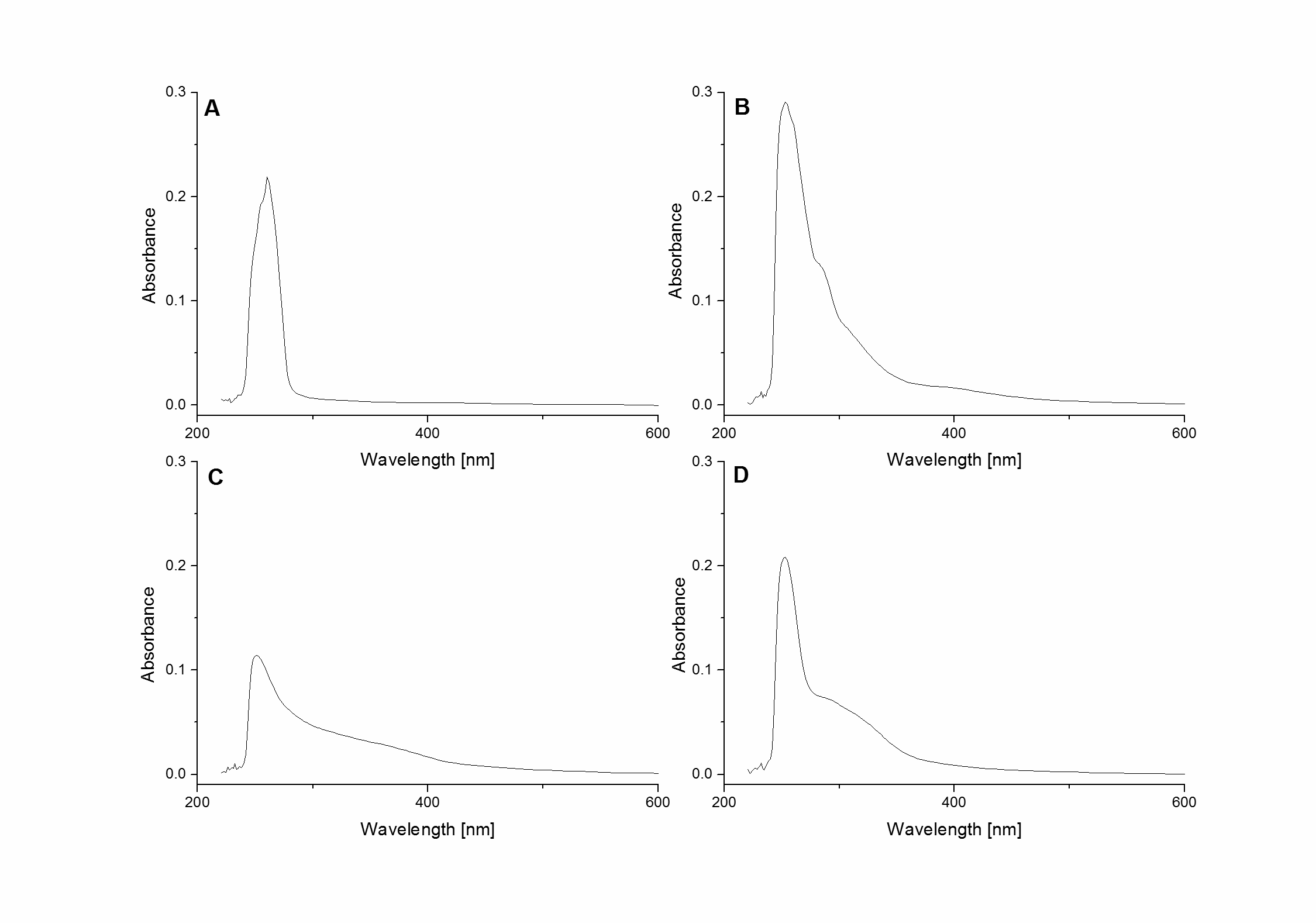

Supplement: S2 Fig — (BMP) [file pone.0131773.s002.bmp]
