# Supplementary material for: Potential of Wood-Rotting Fungi to Attack Polystyrene Sulfonate and Its Depolymerisation by Gloeophyllum trabeum via Hydroquinone-Driven Fenton Chemistry
Source: PLoS One. 2015 Jul 6;10(7):e0131773. doi: 10.1371/journal.pone.0131773 (PMC4493105; doi:10.1371/journal.pone.0131773)
Supplement: S1 File — (DOCX) [file pone.0131773.s003.docx]

S1 Supporting Information – Supporting MS data

**Title: Potential of Wood-rotting Fungi to Degrade Polystyrene Sulfonate and its Depolymerisation by *Gloeophyllum trabeum* via Hydroquinone-Driven Fenton Chemistry**

Authors: Martin C. Krueger^1^, Ulrike Hofmann^1^, Monika Moeder^2^ and Dietmar Schlosser^1^*

^1^ Department Environmental Microbiology, Helmholtz Centre for Environmental Research - UFZ, Leipzig, Germany

^2^ Department Analytical Chemistry, Helmholtz Centre for Environmental Research - UFZ, Leipzig, Germany

**Number of pages: 7**

**Content:**

1. Identification of the products determined in the sample extracts

1.1. Experimental conditions for the gas chromatographic-mass spectral analysis (GC-MS)

2. GC-MS analysis of the reference 2,5-DMHQ

3. Chromatograms of the GC-MS analysis of the chloroform extract of a sample after

10 days of incubation

4. Mass spectral characteristics of the signals detected

5. Figures A-E

1. **Identification of the products determined in the sample extracts**
   1. *Experimental conditions for the gas chromatographic-mass spectral analysis (GC-MS)*

GC-MS was applied after extraction of 1.5 ml culture supernatant from day 10 with 3 x 200 µL chloroform (GC-grade, Sigma Aldrich, Seelze, Germany). The extracts were unified and evaporated at a gentle stream of nitrogen gas to a final volume of 1 mL. For analysis, 1 µL were injected splitless (1 min vent time). The analysis were performed with a GC-MSD system (Agilent Technologies, Waldbronn, Germany) equipped with a HP 5MS capillary (30 m x 025 mm i.d., 25 mm film thickness Agilent Technologies). The oven program used following parameters: initially 60 °C - 2 min - 8 K/min - 280 °C - 2.5 K/min - 300 °C. Helium was applied as carrier gas at a flow of 1 mL/min. Injector temperature was set at 280 °C as the transfer line to the mass spectrometer. The mass spectra were acquired at full scan mode in the mass range from 50 u to 500 u. Electron ionization (70 eV) was applied at an ion source temperature of 230 °C. For determining the instrumental detection limit and recoveries of the extraction, the mass detection used selected ion monitoring (SIM) mode with the target ions at m/z 155, 170, 127, 112 (DMHQ) and 69, 139, 95, 153 (DMBQ).

The detection limit of the instrumental method was determined at 0.8 µg/mL and a precision of ± 2.4 %.

The mean recovery of the extraction process for 2,5-DMBQ with chloroform was 88 % (± 7% , n=3) and 65 % for 2,5-DMHQ (n=1).

**2. GC-MS analysis of the reference compound 2,5-DMHQ**

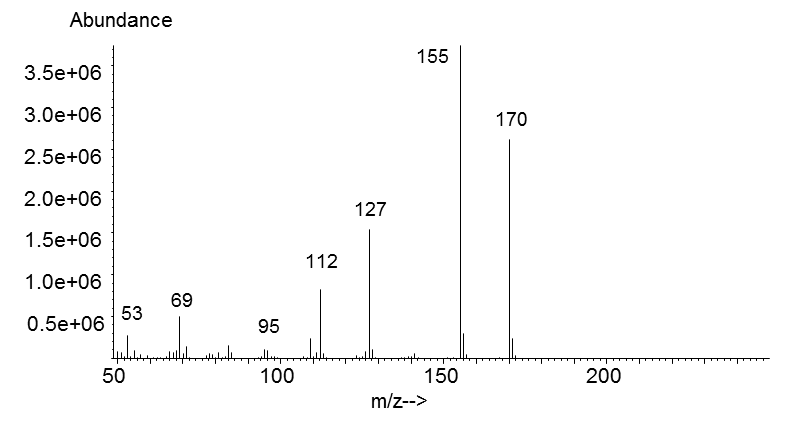


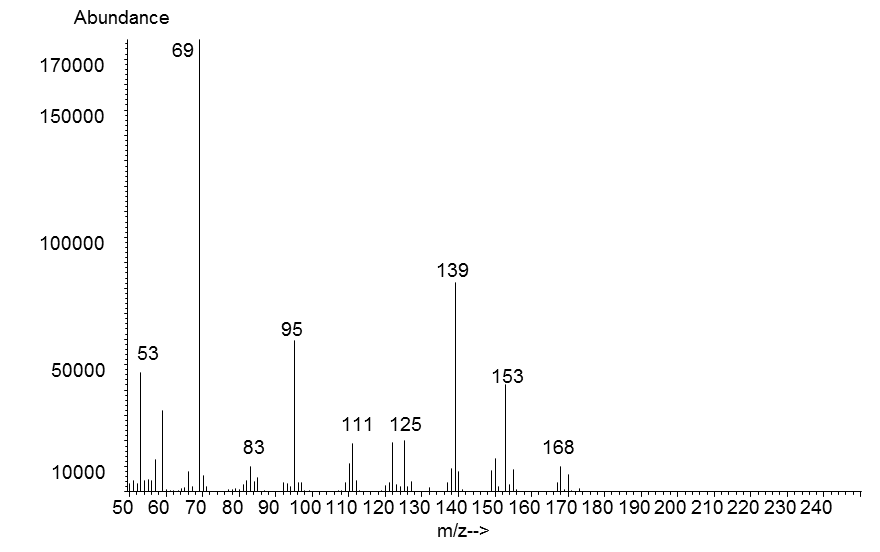


Mass spectrum at 14.92 min

2,5-DMHQ

Mass spectrum at 16.94 min

2,5-DMBQ

**Figure A:** Total ion current chromatogram of the GC-MS analysis of the reference compound generated from 2,5-DMBQ, corresponding mass spectra representing the 2,5-DMHQ and the 2,5-DMBQ remained.

**3. Chromatograms of the GC-MS analysis of the chloroform extract of a sample after 10 days of incubation**

a)

b)

2,5 DMHQ- monoacetate

13.50

14.00

14.50

15.00

15.50

16.00

16.50

17.00

17.50

18.00

18.50

19.00

19.50

20.00

50

100

150

200

250

300

350

400

450

500

Retention time (min)

Abundance

2,5 DMHQ

15.00

16.00

17.00

18.00

19.00

20.00

21.00

1000

2000

3000

4000

5000

Abundance

**1**

**2**

**?**

**3**

Retention time (min)

**Figure B:** GC-MS (SIM) analysis. Ion trace chromatograms of m/z 170: a) of the sample extract (10^th^ d of incubation) and b) of 2,5 DMHQ derivatised with acetic anhydride to mono acetate (**2**) and the diacetate derivative (**3**). Peak **1** represents underivatised 2,5 DMHQ.

The peak at retention time 17.3 min (**?**) was not identified but was also not present in the extracts as the diacetate that was not detected in the sample extracts (3 different samples were analysed).

**4. Mass spectral characteristics of the signals detected**

[M]٠^+^

50

60

70

80

90

100

110

120

130

140

150

160

170

180

190

200

0

200

400

600

800

1000

1200

1400

1600

1800

2000

m/z-->

Abundance

Scan 1942 (15.144 min): 08091442.D (-1881) (-)

155

170

127

69

112

95

60

83

192

146

[M-CH_3_]^+^

**Figure C:** Mass spectrum of the 2,5-DMHQ reference compound with a retention time at 15.14 min.

a)

b)

60

70

80

90

100

110

120

130

140

150

160

170

180

190

200

100

200

300

400

500

600

700

m/z-->

Abundance

Scan 2190 (16.662 min): 08091444.D (-2144) (-1785) (-)

155

170

139

115

95

59

125

66

177

131

81

197

205

185

74

102

[M-COCH_2_]^+^

[M-CH_3_-COCH_2_]^+^

**Figure D:** a) background subtracted mass spectrum of 2,5-DMHQ monoacetate detected at 16.66 min retention time in the GC-MS analysis of the sample extract (10 d incubation), b) mass spectrum of the 2,5-DMHQ monoacetate reference synthesized from 2,5-DMHQ with acetic anhydrate, retention time of 16.52 min.

a)

b)

**Figure E:** a) Mass spectrum of the compound detected at retention time 18.24 min in the sample extract (10 d incubation), b) the mass spectrum of 1-[5-(2-furanylmethyl)-2-furanyl]-ethanone (NIST02 spectral library, 77 % fit).
